# Supplementary material for: Identification of photoperiod-induced specific miRNAs in the adrenal glands of Sunite sheep (Ovis aries)
Source: Front Vet Sci. 2022 Jul 22;9:888207. doi: 10.3389/fvets.2022.888207 (PMC9354845; doi:10.3389/fvets.2022.888207)
Supplement: Supplementary file 1 [file Data_Sheet_1.ZIP › Supplementary file/miRDeep2 file.zip!/miRDeep2 file/Novel_Structure/LP42A3/Novel_666.pdf]

Provisional ID	:	NC_019458.2_876
Score total	:	1.4
Score for star read(s)	:	-1.3
Score for read counts	:	0
Score for mfe	:	1.1
Score for randfold	:	1.6
Score for cons. seed	:	
Total read count	:	2401
Mature read count	:	2401
Loop read count	:	0
Star read count	:	0

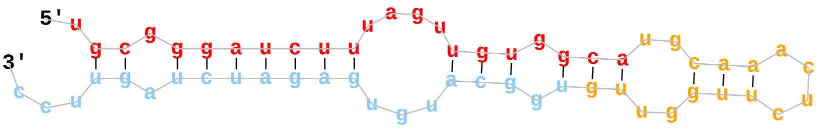

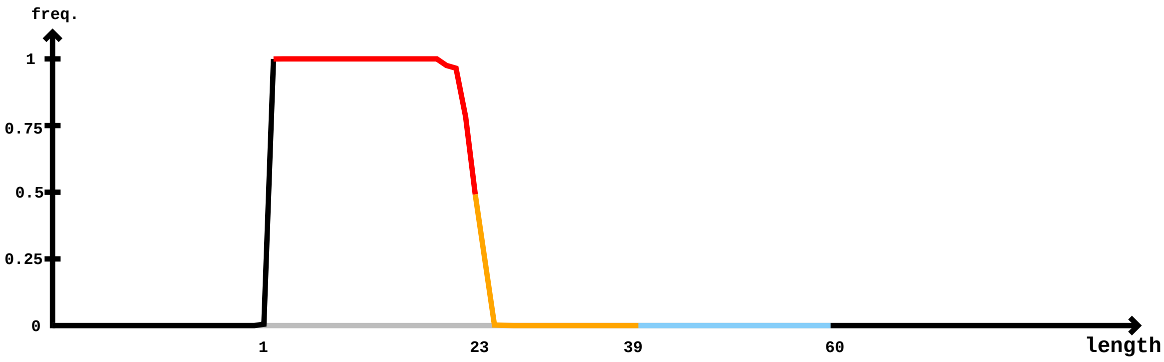

Mature **Star**

```
5'- ucgucagucuuuguuuugggu ugcgggaucuuuaguuguggca ugcaaacucuugguug uggcaugugagaucuaguucc ccaacaagggauggaugcuuacuccuuggaa -3'    exp
    .((((.((((((...(((((..((.(((((((....(((.(((..(((....)))..))).)))...))))))).))..)))))..)))))).))))..............                reads   mm   sample
    ...................uugcgggaucuuuaguugugg.......................................................................                 1       0    seq
    ...................Augcgggaucuuuaguuguggca.....................................................................                 10      1    seq
    ...................uugcgggaucuuuaguuguggca.....................................................................                 1       0    seq
    ....................ugcgggaucuuuaguugu.........................................................................                 51      0    seq
    ....................ugcAggaucuuuaguugu.........................................................................                 2       1    seq
    ....................ugcgggaucuuuaguugA.........................................................................                 2       1    seq
    ....................ugcgggaucuuuaguugC.........................................................................                 4       1    seq
    ....................uAcgggaucuuuaguugug........................................................................                 3       1    seq
    ....................ugcgggaucuuuaguuguA........................................................................                 22      1    seq
    ....................ugcgggaucuuuaguugugU.......................................................................                 32      1    seq
    ....................ugcgggaucuuuaguugugA.......................................................................                 122     1    seq
    ....................ugcAggaucuuuaguugugg.......................................................................                 3       1    seq
    ....................ugcgggaucuuuagCugugg.......................................................................                 1       1    seq
    ....................ugcgggaucuuuaguugugg.......................................................................                 277     0    seq
    ....................ugcgggaucuuuaguugCgg.......................................................................                 1       1    seq
    ....................ugcgggaucuuuaguuguAg.......................................................................                 1       1    seq
    ....................uAcgggaucuuuaguuguggc......................................................................                 5       1    seq
    ....................ugcgggaucuuuaguugugAc......................................................................                 3       1    seq
    ....................ugcgggaucuuuaguuguggc......................................................................                 681     0    seq
    ....................ugcgggaucuuuagCuguggc......................................................................                 1       1    seq
    ....................ugcAggaucuuuaguuguggc......................................................................                 7       1    seq
    ....................ugcUggaucuuuaguuguggca.....................................................................                 1       1    seq
    ....................ugcgggaucuuuaguugCggca.....................................................................                 2       1    seq
    ....................ugcgAgaucuuuaguuguggca.....................................................................                 7       1    seq
    ....................ugcgggaucuuuaguugugUca.....................................................................                 1       1    seq
    ....................ugcgggaucuuuaguuguggca.....................................................................                 1106    0    seq
    ....................uAcgggaucuuuaguuguggca.....................................................................                 5       1    seq
    ....................ugcgggaucuuuaguugugAca.....................................................................                 1       1    seq
    ....................ugcgggaucuuuaguugAggca.....................................................................                 1       1    seq
    ....................ugUgggaucuuuaguuguggca.....................................................................                 4       1    seq
    ....................ugcgggaucuuuaguuguggUa.....................................................................                 18      1    seq
    ....................ugcgggaucuuuaguuguggcaC....................................................................                 18      1    seq
    ....................ugUgggaucuuuaguuguggcau....................................................................                 1       1    seq
    ....................ugcgggaucuuuaguuguggcauA...................................................................                 2       1    seq
```

```
                     Mature                                Star
ucgucagucuuuguuuuggguugcgggaucuuuaguuguggcaugcaaacucuugguuguggcaugugagaucuaguuccccaacaagggauggaugcuuacuccuuggaa
.....................ugcgggaucuuuaguuguggcauC..................................................................    1    1    seq
.....................ugcgggaucuuuaguuguggcaugc.................................................................    1    0    seq
.....................ugcgggaucuuuaguuguggcaugU.................................................................    1    1    seq
......................gcgggaucuuuaguuguggc.....................................................................    1    0    seq
```
